# Resources for methylome analysis suitable for gene knockout studies of potential epigenome modifiers

**DOI:** 10.1186/2047-217X-1-3

**Published:** 2012-07-12

**Authors:** Gareth A Wilson, Pawandeep Dhami, Andrew Feber, Daniel Cortázar, Yuka Suzuki, Reiner Schulz, Primo Schär, Stephan Beck

**Affiliations:** 1 Medical Genomics, UCL Cancer Institute, University College London, London, UK; 2 Institute of Biochemistry and Genetics, Department of Biomedicine, University of Basel, Basel, Switzerland; 3 Department of Medical and Molecular Genetics, King’s College London, London, UK

**Keywords:** Methylome, MeDIP-seq, Epigenetics, Epigenomics, DNA methylation, Computational pipeline, MeDUSA

## Abstract

**Background:**

Methylated DNA immunoprecipitation (MeDIP) is a popular enrichment based method and can be combined with sequencing (termed MeDIP-seq) to interrogate the methylation status of cytosines across entire genomes. However, quality control and analysis of MeDIP-seq data have remained to be a challenge.

**Results:**

We report genome-wide DNA methylation profiles of wild type (wt) and mutant mouse cells, comprising 3 biological replicates of Thymine DNA glycosylase (*Tdg*) knockout (KO) embryonic stem cells (ESCs), *in vitro* differentiated neural precursor cells (NPCs) and embryonic fibroblasts (MEFs). The resulting 18 methylomes were analysed with MeDUSA (Methylated DNA Utility for Sequence Analysis), a novel MeDIP-seq computational analysis pipeline for the identification of differentially methylated regions (DMRs). The observed increase of hypermethylation in MEF promoter-associated CpG islands supports a previously proposed role for *Tdg* in the protection of regulatory regions from epigenetic silencing. Further analysis of genes and regions associated with the DMRs by gene ontology, pathway, and ChIP analyses revealed further insights into *Tdg* function, including an association of TDG with low-methylated distal regulatory regions.

**Conclusions:**

We demonstrate that MeDUSA is able to detect both large-scale changes between cells from different stages of differentiation and also small but significant changes between the methylomes of cells that only differ in the KO of a single gene. These changes were validated utilising publicly available datasets and confirm *TDG's* function in the protection of regulatory regions from epigenetic silencing.

## Background

DNA methylation is an important epigenetic modification, playing a vital role in genome dynamics. In conjunction with histone modifications, remodeling complexes and non-coding RNAs, it modulates chromatin density and thereby accessibility of the underlying DNA to the transcriptional machinery. As a result, DNA methylation is involved in a diverse range of processes including embryogenesis, genomic imprinting, cellular differentiation, DNA-protein interactions, and gene regulation [1].

In mammalian genomes, methylation predominantly occurs symmetrically on both DNA strands at palindromic CpG dinucleotides, but the preference between CpG and non-CpG methylation appears to vary with the degree of cell differentiation [2]. Of the methylcytosines detected in human somatic cells (fetal lung fibroblasts), more than 99% have been shown to be in a CpG context. In contrast, in embryonic stem cells there is abundant methylation in non-CpG contexts, comprising approximately 25% of the total number of methylcytosines detected [3].

There are a plethora of methods available for the exploration of DNA methylation [4,5]. Since the advent of high throughput sequencing, methods for genome-wide methylome profiling are both available and increasingly affordable. Methylated DNA immunoprecipitation (MeDIP) [6] is a popular enrichment based method, in which an antibody capable of recognizing 5-methylcytosine (5mC) is utilised to immunoprecipitate the methylated fraction of the genome. A number of tools have been developed for the analysis of MeDIP data, including Batman [7], MEDME [8], MEDIPS [9], MeQA [10], and SeqMonk [11]. MeDIP, originally developed for use on arrays, can be combined with sequencing (termed MeDIP-seq) to interrogate the methylation status of cytosines across entire genomes. MeDIP-seq has been used in numerous studies, including the first mammalian methylome [7] and the first cancer methylome [12].

Thymine DNA glycosylase (TDG), a member of the uracil DNA glycosylase (UDG) superfamily of DNA repair enzymes, has been shown to be essential for embryonic development [13]. However, its exact functionality is still unclear. The protein structure and biochemical properties suggest it has a role in DNA repair, whilst interactions with other proteins indicate involvement in the regulation of gene expression [14]. A recent study has shown TDG to have a dual role in epigenetic maintenance. Firstly, as a structural component, TDG is involved in the maintenance of active and bivalent chromatin through interactions with activating histone modifiers. Secondly, TDG appears to provide DNA repair functionality leading to the ability to erase aberrant methylation at GC-rich promoter regions. This dual-role suggests that TDG is important for the protection of critical genomic regions from *de-novo* DNA methylation and heterochromatinization during development [13,15].

## Data description

Here, we present a comprehensive resource comprising data and tools for the study of genome-wide methylation profiles in mouse. 18 methylomes were generated using a dataset of over 251 million uniquely mapped fragments (>502 million mapped paired-end reads) and were processed using our novel MeDIP-seq computational analysis pipeline (Methylated DNA Utility for Sequence Analysis, or MeDUSA). The methylomes represent 6 biological cohorts, demonstrating robust detection of differentially methylated regions (DMRs) in the context of both differentiation and, more subtly, a gene KO system, in this case *Tdg*. Further analysis of these DMRs by integration with Chromatin Immunoprecipitation (ChIP) data provides new insights into the functionality of TDG.

The MeDIP-seq data from this study have been submitted to the NCBI Gene Expression Omnibus [16] under accession no. GSE27468. Wig tracks displaying normalised read depth can be accessed through the Ensembl HEROIC portal [17] or http://www2.cancer.ucl.ac.uk/medicalgenomics/tdg_web/trackList.php. MeDUSA can be downloaded from our MeDUSA homepage [18]. All supporting data and associated files from the MeDUSA pipeline are also available from *GigaScience*[19].

## Analysis

### Genome-wide mapping of 18 mouse methylomes

MeDIP-seq was performed, as described in Feber *et al.*[12], on 18 samples, representing 6 biological cohorts. 6 samples were derived from mouse embryonic stem cells (ESCs) (3 *Tdg*^*+/−*^*,* 3 *Tdg*^*−/−*^*),* 6 samples were from mouse neural precursor cells (NPCs) (3 *Tdg*^+/−^, 3 *Tdg*^−/−^), and 6 samples were obtained from mouse embryonic fibroblasts (MEFs) (3 *Tdg*^+/+^, 3 *Tdg*^−/−^). The biological samples were generated as described by Cortázar *et al.*[13].

Over 500 million reads were uniquely mapped to the reference genome (NCBIM37), using BWA [20] (alignment score > = 10), representing over 250 million mapped fragments (Additional file 1: Table S1). Additionally, fragment length normalisation was performed in order to eliminate potential bias in coverage resulting from discrepancies in the distribution of fragment lengths between samples. Correlation in genome-wide sequence coverage between replicates was calculated. The correlation between the 3 biological replicates of the NPC and MEF cohorts was high (>0.83 and >0.90 respectively) (Additional file 1: Table S2). Correlation in the ESC cohorts was considerably lower (>0.51), perhaps reflecting greater epigenetic dynamism in the undifferentiated cells. Non-CpG methylation, believed to be prevalent in undifferentiated cells, has been shown to display both lower methylation levels within a cell population and lower conservation between cell lines [21,22]. If true, this dynamism would not be seen in technologies such as MethylCap [23] that only pull back methylation from CpG dinucleotides, and could potentially contribute to the increased variation between ESC replicates. Correlation between ESC samples in CpG islands was notably higher (0.85-0.89). Whilst the increased variation in ESCs, reflected by the lower correlation, could present challenges, our method for DMR identification can locate true biological variation whilst minimizing false positives. In addition to determining the correlation between biological replicates, we determined the proportion of CpG sites in the reference genome that were covered by aligned fragments (Additional file 1: Table S1 and Additional file 1: Figure S1). Furthermore, both saturation analysis [9] and between replicate correlations [24] indicated we had sufficient reads to provide reproducible genome-wide methylation profiles (Additional file 1: Table S2). An example of the output from the saturation and coverage analysis performed in the MeDUSA pipeline, by MEDIPS is shown in Additional file 1: Figure 1.

**Figure 1:**
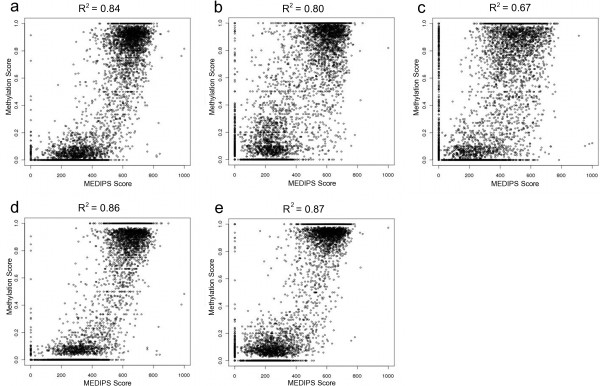
**Comparison of MEDIPS normalised MeDIP-seq data with RRBS**[25]**and BS-seq**[26]**data.(a)** ESC *Tdg*^*+/−*^ MeDIP vs ESC wt RRBS. **(b)** NPC *Tdg*^*+/−*^ MeDIP vs NPC wt RRBS. **(c)** MEF wt MeDIP vs MEF wt RRBS. **(d)** ESC *Tdg*^*+/−*^ MeDIP vs ESC wt BS-seq. **(e)** NPC *Tdg*^*+/−*^ MeDIP vs NPC wt BS-seq.

We validated our MeDIP-seq results, utilising previously published reduced representation bisulphite sequencing (RRBS) data from wild type (wt) ESCs, wt NPCs and wt MEFs [25], and whole-genome bisulphite sequencing (BS-seq) of wt ESC and wt NPC [26]. For the purpose of validation, absolute methylation values were calculated from the MeDIP read counts for the ESC *Tdg*^*+/−*^, NPC *Tdg*^*+/−*^ and the MEF *Tdg*^*+/+*^ cohorts using MEDIPS [9]. Reads from each of the replicates within each cohort were merged into a single cohort-specific dataset. Validation was performed for all CpG sites in ESC and NPC that were covered (minimum depth of 10) in both the RRBS and the BS-seq datasets. Only RRBS data was available for MEFs. Overall correlation was high between the data types, ranging from 0.86 for the ESC comparison, to 0.80 for the NPCs and 0.67 for the MEFs. This validation also supported our saturation analysis, as regions lacking coverage in MeDIP-seq reads were shown to be largely unmethylated as opposed to being an artifact resulting from insufficient sequencing (Figure 1). The decrease in correlation as the cells become increasingly differentiated could be an artifact of the CpG subset analysed, though it may also reflect true clonal effects. This is supported by the decrease in correlation between the RRBS and Bis-seq data for ESC (0.96) and NPC (0.86).

Our dataset can be accessed through Ensembl [27] as part of the HEROIC portal [17] (Additional file 1: Figure S2) and the *GigaScience* database [19].

### MeDUSA computational pipeline

The MeDIP-seq data were processed using our novel analysis pipeline MeDUSA (Methylated DNA Utility for Sequence Analysis). MeDUSA brings together numerous software packages to perform a full analysis of MeDIP-seq data, including sequence alignment, quality control (QC), and determination and annotation of DMRs. In contrast to previously published tools for MeDIP-seq analysis (e.g. Batman [7], MEDIPS [9]) in which the primary focus was the ability to accurately call absolute methylation values based on CpG density, the focus for MeDUSA is the accurate and statistically rigorous identification of DMRs. To achieve this, relative changes in DNA methylation between cohorts (rather than absolute changes within cohort) need to be determined, and as such the problem has much in common with identifying differential expression between RNAseq cohorts. MeDUSA utilises several applications from within the USeq software suite [24], and in turn uses the R Bioconductor [28] package DESeq [29] for differential count analysis. In addition, MeDUSA controls several other important functions from the alignment (BWA [16]) and subsequent filtering (SAMtools [30]) through the generation of numerous quality control metrics (FastQC[31] and MEDIPS [9]), and preliminary annotation of the DMRs (utilising the capabilities of BEDTools [32]).

There are several issues that can hinder MeDIP-seq analysis, particularly when identifying DMRs. Firstly, sequencing depth between samples will vary and so read counts need to be normalised. Whilst global read count normalisation can help address this problem, it does not account for ‘competition’ effects. Such competition can be seen in RNA-seq, in which sample specific highly expressed genes can lead to a depressed normalised read count in other genes and hence a bias when comparing samples [33]. Analogous situations can be found in MeDIP-seq, where sample-specific repeat methylation could potentially bias analyses, particularly given the large proportion of methylated repetitive sequence found in the genome, or samples with high levels of non-CpG methylation could lead to an underestimation of methylation levels at CpG sites. Secondly, MeDIP-seq experiments will often have small numbers of biological replicates, and hence it can be difficult to obtain reliable estimates of model parameters to fit statistical models and locate real differences between samples. MeDUSA utilises DESeq to address these challenges. DESeq estimates variance in a local fashion and in doing so removes potential selection biases [29]. Additionally, rather than attempting to reliably estimate the variance and mean parameters of the distribution from limited numbers of replicates, DESeq estimates a more flexible, mean-dependent local regression. Typically, there is enough data available in these experiments to allow for sufficiently precise local estimation of the dispersion [29] and hence avoid bias towards certain areas of the dynamic range when identifying DMRs. Finally, it is possible that differences in DNA fragment size distributions between samples could compromise accurate biological interpretation. MeDUSA provides the option to perform fragment length normalisation through read sub-sampling to equalize the distributions, thus eliminating this potential bias.

**Figure 2:**
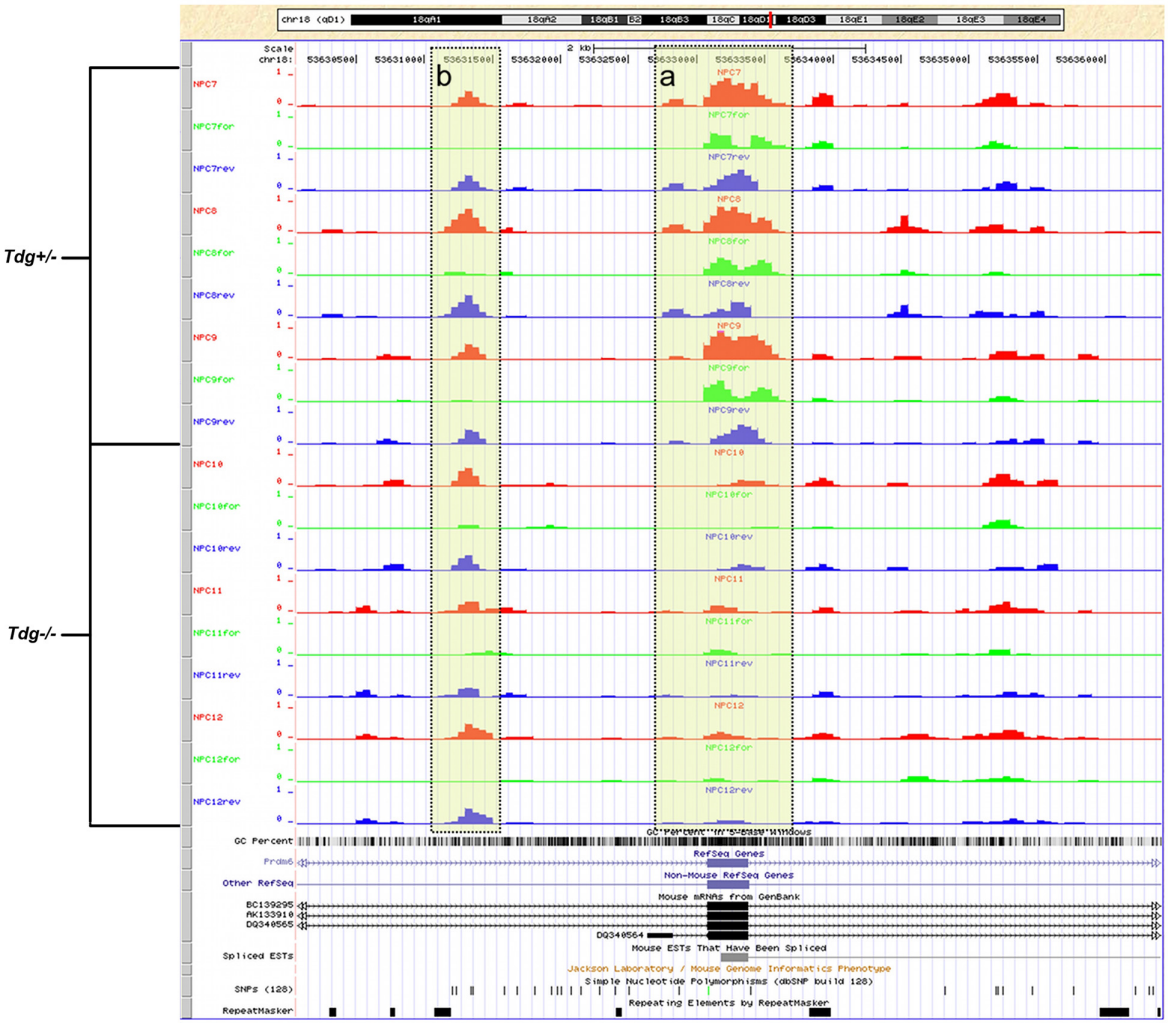
**Mouse NPC methylomes viewed in the UCSC browser.** Each sample has 3 tracks representing total coverage (red) and coverage on forward (green) and reverse (blue) strands. The data range on the Y-axis of each track represents the read depth in reads per million (RPM). Box a highlights a *Tdg*^*−/−*^ hypomethylated DMR in exon 2 of *Prdm6*. Box b indicates an example of potential non-CpG methylation where the methylation signal is driven by a single strand, in this case the reverse strand.

Additionally, taking advantage of the genome-wide nature of MeDIP-seq and the affinity of the MeDIP-seq antibody for methylated cytosine (i.e., not in a CpG-methylation specific context), MeDUSA also allows identification of potential non-CpG methylation [11]. By determining the ratio of fragments originating from each strand, we can infer the strand from which the methylation signal originated. An even distribution on both strands would be anticipated for a methylated region driven by symmetric CpG methylation. In contrast an asymmetric fragment distribution, preferentially aligning to one strand, could indicate the presence of non-CpG methylation, particularly when supported by sequence motif analysis. Compared to previous methods, the MeDUSA-analysed profiles result in 3 separate tracks per methylome, with the proportion of reads indicated that are mapping to both, forward or reverse strands, allowing assessment of CpG and potential non-CpG methylation (Figure 2). The potential to search for DMRs driven by non-CpG methylation illustrates the flexibility inherent when performing a relative analysis of MeDIP samples. This flexibility means that the pipeline will also be equipped to analyse enrichment data for other DNA modifications such as hydroxymethylcytosine, formylcytosine and carboxylcytosine.

### Identification of methylation differences associated with differentiation

**Figure 3:**
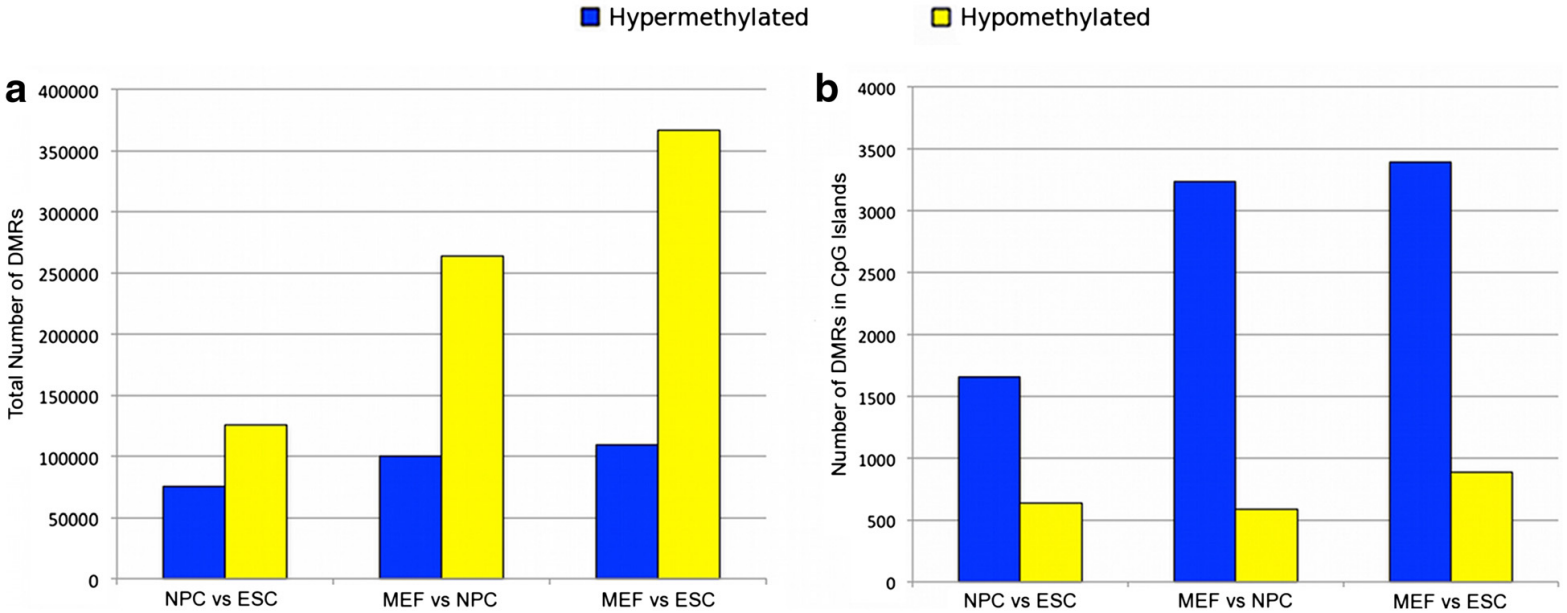
**Number of DMRs identified in comparisons of samples from different stages of differentiation.** DMRs hypermethylated in the more differentiated state are shown in blue, those hypomethylated in the more differentiated state are in yellow. **(a)** All DMRs. **(b)** DMRs located in CpG Island regions.

MeDUSA utlises USeq MultipleReplicaScanSeqs [24] and the DESeq R Bioconductor package [29] to locate statistically significant DMRs. Using MeDUSA, we compared the MeDIP-seq methylation profiles of ESC *Tdg*^*+/−*^, NPC *Tdg*^*+/−*^ and the MEF *Tdg*^*+/+*^ samples to define large numbers of statistically significant DMRs associated with different stages of differentiation. As expected, more DMRs (maximum FDR 5%) were found between ESCs and MEFs (366,980 hypomethylated in MEFs, 109,694 hypermethylated in MEFs) than between ESCs and NPCs (125,335 hypomethylated in NPCs, 75,496 hypermethylated in NPCs) or NPCs and MEFs (263,911 hypomethylated in MEFs, 100,365 hypermethylated in MEFs). DMRs ranged in size from 29 bp to 46,820 bp (Additional file 1: Figure S3a). The distance between adjacent DMRs was largely dependent on total number of DMRs identified (correlation = −0.92) and ranged from 500 bp to 7,501,000 bp (Additional file 1: Figure S3b). Comparison of global methylation status suggested a trend for decreased methylation during differentiation (shown by the increased numbers of hypomethylated DMRs versus the numbers of hypermethylated) (Figure 3a). This is supported by data from previous studies of human cells [2,3]. Of the 125,335 hypomethylated DMRs found between NPCs and ESCs, 85% were also deemed to be hypomethylated between MEFs and ESCs (Additional file 1: Figure S4). Additionally, 31% underwent further hypomethylation between the NPC and MEF state, illustrating that in some cases hypomethylation is a continuous process through multiple stages of differentiation. A contrast to this global hypomethylation with differentiation was shown in CpG island regions. We saw more instances of increased methylation in CpG islands along the transition to differentiated cells (*p*-value = < 0.001) (Figure 3b). This supports the idea of an increasingly restrictive pattern of gene expression associated with differentiation [2]. These dynamic islands include many regions associated with system development, including numerous members of the *HoxPouSixKlf*, and *Tcf* gene families. Enrichment analysis of genes associated with these islands shows significant enrichment in roles for tissue development (*p*-value = 2.02e-56) and embryonic development (*p*-value = 8.11e-43). Additionally, as expected, the Homeobox domain was found to be strongly associated within these island regions (FDR = 4.85e-32).

**Figure 4:**
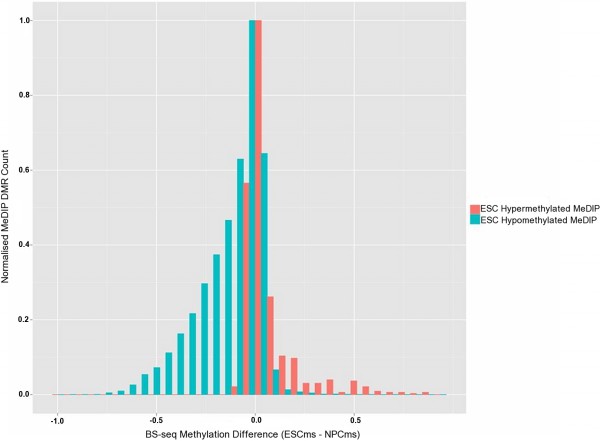
**Difference in methylation score (ESCms – NPCms) calculated from BS-seq data**[26]**for each DMR called from the MeDIP cohorts.** Regions displaying ESC hypermethylation in the BS-seq data will score >0, those displaying hypomethylation will score <0. Frequency of hypermethylated MeDIP DMRs are shown in red, hypomethylated DMRs in blue, indicating the concordance between datasets.

To perform a large-scale validation of all the DMRs (maximum FDR 1%) called between ESC and NPC cohorts, BS-seq data [26] was utilised (Figure 4). The methylation score for each CpG dinucleotide was determined from the BS-seq data. The NPC methylation score (NPCms) was subtracted from the ESC methylation score (ESCms) to determine the difference in methylation for each CpG. For each MeDIP DMR, the overall methylation change (Δms) was calculated. Of the 16,592 hypomethylated MeDIP ESC DMRs tested, 13,644 showed decreased methylation (ESCms – NPCms = Δms < 0) in the BS-seq data. 7,884 showed a Δms of < −0.1, in contrast only 147 showed Δms >0.1. Similarly, of the 738 hypermethylated ESC DMRs tested, 545 also showed increased ESC CpG methylation in the BS-seq data (ESCms – NPCms = Δms > 0). Of these, 246 had Δms >0.1, compared with only 2 with Δms < −0.1. According to this analysis, 82% of the called DMRs are supported by independent data (*p*-value < 0.001).

### Tdg KO-associated differences in methylation

Having demonstrated the ability to call DMRs between cohorts expected to have large numbers of DMRs, we used the MeDUSA pipeline to try and identify DMRs between cohorts expected to have small numbers of significant DMRs using MEFs wild type and single gene (*Tdg*) knockout. By comparing cohorts from within the same differentiation state, the effect of the absence of TDG on the global methylation profile could be explored. DMRs were called for each cell type with a maximum false discovery rate (FDR) of 5%. Using this approach we identified 32,975 (13,590 hypermethylated in *Tdg*^−/−^, 19,385 hypomethylated in *Tdg*^*−/−*^) DMRs in MEFs (Additional file 1: Figure 5Sa), 942 (609 hypermethylated in *Tdg*^*−/−*^, 333 hypomethylated in *Tdg*^*−/−*^) in NPCs (Additional file 1: Figure 5Sb), and 0 in ESCs. Whilst attempts to locate DMRs between the ESCs may have been restricted by the increased background variability in the undifferentiated cells (intra-cohort mean correlation = 0.56), these data suggest that the direct impact on methylation of loss of TDG is greater in more differentiated cell types.

**Figure 5:**
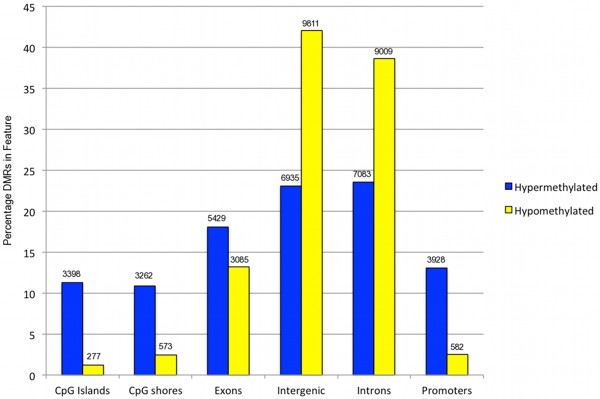
**Proportion of DMRs found in different genomic features.** DMRs hypermethylated in MEF *Tdg*^*−/−*^ are shown in blue, those hypomethylated in MEF *Tdg*^*−/−*^ are in yellow. The numbers represent the amount of DMRs found in each feature type.

Figure 5 shows the proportion of DMRs found in different genomic features within the MEF comparison. The majority of DMRs were found in intronic (n = 16,092, 44% hypermethylated in *Tdg*^*−/−*^, 56% hypomethylated in *Tdg*^*−/−*^) and intergenic regions (n = 16,746, 41% hypermethylated in *Tdg*^*−/−*^, 59% hypomethylated in *Tdg*^*−/−*^). Of the MEF DMRs found in CpG islands (n = 3,675), the majority were hypermethylated (n = 3,398). This supports the hypothesis that TDG, when recruited to regions of high GC content, protects against de-novo methylation [13]. In the absence of TDG, an increase in methylation in such regions is observed.

### Enrichment analysis of tdg KO-associated DMRs in MEF

To gain preliminary insights into their possible function, the *Tdg* KO-associated MEF DMRs were subjected to further bioinformatic analyses. Using GREAT [34], it was possible to interrogate annotations from 20 different ontologies utilising the genomic coordinates of the DMRs. Hypermethylated DMRs (Additional file 1: Table S3a) were found to be associated with transcription regulation (q-value = <e-300), DNA binding (q-value = <e-300), system development (q-value = <e-300), as too were sequences implicated in the regulation of various metabolic processes (q-value = e < −300). There was strong association with Polycomb targets, specifically H3K27me3-marked genes (q-value = <e-300) and targets of SUZ12 and EED (q-value = <e-300), both of which are key components of the PRC2 complex [35]. Additionally, the significant association with genes expressed during Theiler stage 20 (embryonic day 12) (q-value = <e-300) and stage 17 (embryonic day 10.5) (q-value = e < −300) supports previous work showing that it is at this stage of the development of *Tdg* null embryos when internal haemorrhage is detected [13]. PANTHER Pathway analysis (q-value = 1.52e-33) and MSigDB Pathway analysis (q-value = 1.79e-25) both highlighted genes involved in the Wnt signaling pathway as being significantly associated with hypermethylated MEF DMRs. Additionally, the data were analysed with integrated pathway analysis (IPA, Ingenuity® Systems[36]). IPA Canonical Pathway Analysis also highlighted the Wnt signaling pathway (BH *p*-value = 4.15e-11) (Additional file 1: Figure S6a). This pathway has been shown to be important during cell differentiation and has also been linked with cancer [37]. Cancer related pathways on the whole were also shown to be enriched (q-value = 3.01e-45). Enrichment of the embryonic stem cell pluripotency canonical pathway (human) was also highly significant (BH *p*-value = 8.57e-11) (Additional file 1: Figure S6b). Of the 153 genes involved in this pathway, 78 were associated with a DMR. The signal from the hypomethylated DMRs was less strong than from the hypermethylated (Additional file 1: Table S3b). Interestingly, there was a significant enrichment relating to terms associated with ion channel activity (q-value = 1.97e-46), ion transport (q-value = 1.07e-28) and extracellular structure organization (q-value = 1.22e-38).

Using the resource hmChIP [38], the DMRs were compared to publicly available ChIP (ChIP-chip and ChIP-seq) datasets to determine if significant association existed with specific chromatin marks and transcription factor binding sites. The analysis showed a significant overlap between hypermethylated DMRs and regions marked with H3K27me3 [39] (FDR < e-96) and H3K9me3 [40] (FDR < e-45) in numerous mouse ESC datasets. Additionally, highly significant overlap was seen with occupation by SUZ12 [41] (FDR < e-190), JARID2 [42] (FDR < e-190) and EZH1 [39] (FDR < e-190). As previously noted, SUZ12 is a component of PRC2. JARID2 is an associating partner of PRC2 and facilitates its access to chromatin [43]. EZH1 has also been shown to maintain repressive chromatin [44]. Hypermethylation in these regions in *Tdg*^−/−^ cells supports a possible role of TDG in the protection of polycomb repressed but poised gene promoters from *de-novo* methylation. On the other hand, the hypomethylated DMRs show significant association with regions occupied with the activating histone mark H3K9ac [45] (FDR = 1.90e-165).

### MEF DMRs preferentially locate in low-methylated regions (LMRs)

**Figure 6:**
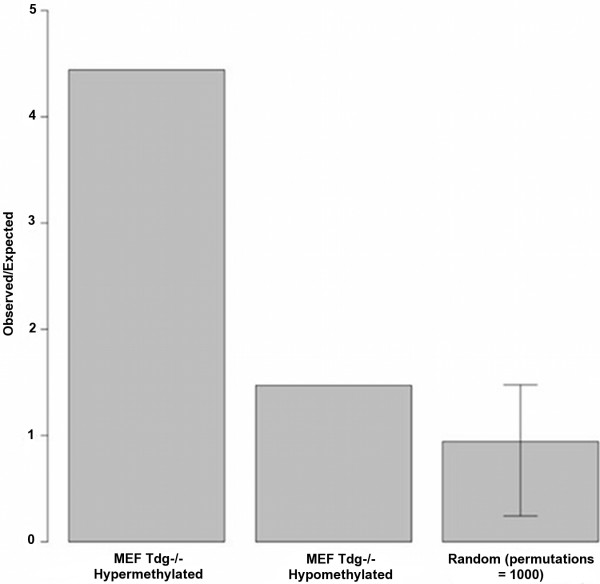
**Overlap between MEF*Tdg***^***−/−***^**DMRs and LMRs**[26]**represented by Observed/Expected ratio.** Data for randomly selected genomic regions also shown, bars indicate the maximum and minimum ratio achieved from 1,000 permutations.

Low-methylated regions (LMRs) have recently been identified as a distinct genomic feature capable of performing as CpG poor, distal regulatory regions [26]. These regions form dynamically through the binding of transcription factors. Once the transcription factor is bound, demethylation follows. The evidence for TDG transcription factor interactions, coupled with its ability to maintain CpGs in an unmethylated state suggests a potential role for TDG in the formation or maintenance of these regions. In the absence of TDG ChIP-seq data, we sought to identify regions of overlap between LMRs and DMRs. A significant number of MEF *Tdg*^*−/−*^ hypermethylated DMRs located in LMR regions (*p* < 0.001) (Figure 6), supporting a prospective role for TDG in LMR formation. Interestingly, this association was found despite comparing NPC LMRs to MEF DMRs. LMRs are reported to be dynamically formed during differentiation and only a small fraction are shared between ESCs and NPCs [26]. Surprisingly, significant association was not found between LMRs and hypermethylated NPC *Tdg*^*−/−*^ DMRs. Further work is required to elucidate the significance of these associations.

## Discussion

Here we report a murine methylome resource, which is publicly accessible to the wider research community through a dedicated Ensembl portal. All 18 methylomes are available to be viewed in their genomic context or downloaded for further analysis. The resource includes the ability to view strand-specific methylation changes, allowing inference of respective signal contributions from CpG and/or potential non-CpG methylation. This property is of particular use when interrogating stem cell datasets in which non-CpG methylation is reportedly prevalent. Additionally, the development of the MeDUSA pipeline allowed for the analysis of the MeDIP-seq data from alignment and QC through to calling and annotation of significant DMRs. MeDUSA does not seek to replace existing tools that generate absolute methylation profiles, instead, through pipelining currently available software, it quickly and easily facilitates to study relevant biological questions that researchers may have concerning their specific cohorts. MeDUSA is easily customizable and can be easily extended with additional applications.

Using *Tdg* wt and mutant cells as example we demonstrate the utility of MeDUSA for detecting small but significant DMRs in KO studies. As predicted from previous observations [13], the number of KO-associated DMRs increased with increasing differentiation. By performing a range of computational analyses, we were able to consistently link *Tdg* KO-associated DMRs with regulation of transcription during developmental processes and regions involved in the maintenance of repressive chromatin, particularly those occupied with PRC2. These findings support the notion that TDG may be involved in the protection of critical regions from *de-novo* methylation by actively demethylating erroneously methylated cytosines. Recent studies have shown that TET catalyses the oxidation of 5-methylcytosine (5mC) to 5-hydroxymethylcytosine (5hmC), 5-formylcytosine (5fC) and 5-carboxycytosine (5caC), the latter two being substrates for TDG and, thus, readily replaced by unmodified cytosine via base excision repair (BER) [46,47]. Additionally, we linked *Tdg* KO-associated DMRs in MEFs with distal regulatory low-methylated regions (LMRs), possibly suggesting a role for TDG in the formation of these regions. The potential role of *Tdg* in mediating these changes is subject of on-going studies.

## Methods

### Samples

The *Tdg* KO strategy, cell culture conditions and in-vitro differentiation procedure used to generate the 18 wt and mutant samples analysed here were as described in Cortázar *et al.* (2011) [13].

### MeDIP-seq

5 μg of DNA from each sample was sonicated to between 50 and 350 bp. Sonicated DNA was then subjected to Illumina’s paired-end library preparation and MeDIP enrichment was performed as described previously [12]. Next generation sequencing (37 bp paired-end reads) was performed on the libraries (size-selected to be between 150 and 200 bp) using an Illumina GAIIx for each sample.

### Data analysis

The generated MeDIP-seq data were analysed using our computational pipeline MeDUSA, which constitutes several discrete stages of analysis and is publicly available from our homepage [18] and via *GigaScience*[19]).

#### Sequence alignment, filtering and quality control

Paired end alignment against the mouse genome (Build NCBIM37) was performed using BWA (v0.5.8) [20] with default settings. Initial filtering to remove those reads failing to map as a proper pair was performed using SAMtools (v0.1.9) [30]. Further filtering removed pairs in which neither read scored an alignment score > =10. Additionally, for each group of non-unique reads (i.e., reads aligned to the exact same start and stop position on the same chromosome), all but one read were discarded. The filtered paired reads were written to file in bed format, where each line represented a uniquely mapping sequenced DNA fragment. This filtering was performed using a custom perl script.

The Bioconductor (v2.7) [28] package MeDIPs (v1.0.0) [9] was used to normalise for size of the sequence library, done by calculating reads per million (RPM) in tiled windows across the genome. Significant difference in fragment length distributions between samples can in turn lead to artificial variation in read counts between samples. Simply trimming aligned fragments to a pre-determined size will not solve the problem, as the bias will have occurred in the initial MeDIP enrichment. Therefore, to reduce any possible bias caused by difference in fragment lengths between samples, fragment length normalisation was performed using a custom perl script. This method seeks to equalize the fragment length distributions through read sub-sampling. The current method requires the removal of fragments that do not fit the normalised distribution.

Wig tracks, for visualization in genome browsers such as Ensembl [27] and UCSC [48], representing library size normalised alignment were generated using a combination of MEDIPS [9] and custom R scripts. In addition to the total alignment wig track, strand specific wig tracks were also generated, enabling the user to infer whether the MeDIP signal is derived by methylation on the forward and/or reverse strand.

To determine our sequence data was of acceptable quality, the tool FastQC (v0.9.4) [31] was used to generate graphical representations of numerous quality metrics such as per base sequence quality and sequence duplication levels. FastQC utilises the Picard suite of utilities [49]. MEDIPS was used to ascertain the reproducibility and CpG coverage of our samples through performing saturation and coverage analyses. Additionally between replicate genome-wide correlations were calculated using QCSeqs from the Useq package [24]. Correlations were calculated using a window size of 500 bp, increasing in 250 bp increments. A minimum number of 5 reads in a window was required prior to inclusion in the correlation.

#### Identification of differentially methylated regions

The USeq (v6.8) [24] suite of tools, specifically MultipleReplicaScanSeqs (MRSS) and EnrichedRegionMaker, were used to identify DMRs between cohorts. MRSS processes Point data for use in the BioConductor package DESeq [29]. Window size was set at 500. MRSS was run using a depth threshold of 10, meaning only regions with a combined depth between all samples of 10 or more were parsed to DESeq for further analysis. DESeq uses a model based on the negative binomial distribution to analyse count data from high-throughput sequencing projects. Significant regions were passed to EnrichedRegionMaker to determine if multiple regions could be combined to create single larger regions. The area of strongest signal within the region was also identified. Output files displaying potential DMRs were generated at various Benjamini & Hochberg (BH) FDRs. For further downstream analysis we used DMRs with an FDR < =5%.

#### Initial annotation of differentially methylated regions

Output files for further biological interpretation were generated using custom perl scripts and feature annotation files in GFF format. The BEDTools software package [32], specifically intersectBed and windowBed, was used extensively to determine the locality of the DMR regions within different feature types. Metadata describing each DMR (e.g., CpG density, nearest gene, genomic region in which the DMR was found and read count within DMR) was obtained. Additionally, counts of DMRs mapping to specified genomic features were generated.

### MeDIP-seq validation

Our MeDIP-seq data were compared with RRBS data from Meissner *et al.*[25] and BS-seq data from Stadler *et al.*[26] for validation. CpG data for 3 RRBS samples (MEF (GSM278888), NPC p9 (GSM278893), ESC (GSM278905)) were obtained from GEO (accession number GSE11034). liftOver [48] was used to convert the files from NCBIM36 to NCBIM37. The CpG data for 2 BS-seq samples, ES and NP, were obtained from GEO (GSE30202). Only CpGs with coverage depth > =10 (in both RRBS and BS-seq for ESC and NPC) were used for validation. CpG sites were extended to create 500 bp windows. A random subset of 5,000 smoothed CpGs was passed to the MEDIPS Bioconductor package [9] which calculated absolute methylation scores from our MeDIP read files for each of our cohorts. Methylation scores were calculated for each extended CpG site in the validation set using default values.

### DMR validation

DMRs generated from the MeDIP comparison were compared to the ESC and NPC BS-seq data (GEO GSE30202). DMRs were filtered to remove those regions containing <10 CpGs, or overlapping an annotated simple repeat region. This was to remove potential biases caused by the presence of non-CpG methylation in the ESC samples undetectable in the BS-seq CpG methylation files. Only CpGs in the BS-seq data with a read depth of > =10 were included. Additionally, the ESC and NPC BS-seq data were quantile normalised to remove biases caused by potential global hypermethylation in the samples. For each DMR, the methylation score for each CpG within the DMR was determined and the value of the NPC score subtracted from the ESC score. Permutation analysis was performed to calculate empirical *p*-value (permutations = 1,000). The proportion of randomly selected regions deemed hypermethylated or hypomethylated by the BS-seq data was compared to the observed result to determine *p*-value.

### Enrichment analysis of DMRs

GREAT (v1.7.0) [34] and IPA (v9.0) (Ingenuity® Systems [36]) were used for enrichment analysis. The genomic co-ordinates of the DMRs were passed to GREAT via the web interface [50]. The analysis was run using ‘Basal + extension’ method with default proximal distances of 5,000 bp upstream and 1,000 bp downstream. The maximum extension was set at 100 kb.

Unlike GREAT, IPA requires gene identifiers rather than co-ordinates. DMRs were associated with their nearest gene up to a maximum of 10 kb upstream and 5 kb downstream of the gene. Using these associated gene identifiers a core analysis against the Ingenuity knowledgebase (genes only), including both direct and indirect relationships was run. Canonical pathways analysis identified the pathways from the IPA library of canonical pathways that were most significant to the dataset. The significance of the association between the dataset and the canonical pathway was measured in 2 ways. Firstly, a ratio of the number of molecules from the dataset that map to the pathway divided by the total number of molecules that map to the canonical pathway. Secondly, Fisher’s exact test was used to calculate a *p*-value determining the probability that the association between the genes in the dataset and the canonical pathway is explained by chance alone.

The database hmChIP [38] was used for integrating DMRs with publicly available ChIP data. hmChIP contains >2,000 samples from >500 ChIP-seq and ChIP-chip experiments, representing a total of >170 proteins. Using liftOver to convert our DMR co-ordinates from NCBIM37 to NCBIM36, we were able to interrogate the hmChIP database for significant overlap between our DMRs and specific ChIP datasets. The analysis was performed against both available ChIP types (TF or DNA-binding proteins and chromatin modifications) including both ChIP-chip and ChIP-seq datasets. Significant overlaps were determined by calculating a *p*-value based on the ratio of the observed overlap to the expected overlap. An FDR, using the BH procedure, adjusting for multiple tests was also calculated.

### Integration of MEF DMRs in LMRs

NPC LMR coordinates were obtained from Stadler *et al.*[26]. MEF DMRs were intersected with LMR regions and base pair overlap determined. Expected base pair coverage was calculated from total DMR, total LMR and genomic base pair counts. Observed/Expected ratios were determined. Random genomic regions (500 bp, n = 1,000-15,000) were analysed in a similar manner. 1,000 permutations of the random data were performed; from this an empirical *p*-value could be calculated.

## Availability of supporting data

The dataset supporting the results of this article is available in the Gene Expression Omnibus repository, GSE27468, and the *GigaScience* database [19].

## Competing interests

The authors declare that they have no competing interests.

## Authors’ contributions

GAW and SB conceived and designed the analyses. PD, AF and YS performed experimental work. GAW analysed the data. GAW, DC, RS and PS contributed reagents, materials or analysis tools. GAW and SB wrote the paper. All authors read and approved the final manuscript.

## Supplementary Material

Additional file 1 Figure S1. Example of saturation and coverage analysis, performed in MeDUSA using the MEDIPS bioconductor package. a) Saturation analysis for ESC1 b) Coverage analysis for ESC1. Additional file 1: Figure S2. Methylomes available through Ensembl (Flicek *et al.* 2011) as part of the EU project HEROIC. Additional file 1: Figure S3. Boxplots displaying a) the DMR size (bp) and b) the genomic distance between DMRs across different cohort comparisons. In b) the width of each box represents the relative number of DMRs in the comparison. Additional file 1: Figure S4: Read density (RPM) at DMRs found between NPC and ESC cohorts. MEF RPM also shown for these sites. Additional file 1: Figure S5. Read density (RPM) at DMRs between a) MEF *Tdg+/+* and MEF *Tdg--‒/--‒* b) NPC *Tdg+/--‒*and NPC *Tdg--‒/--‒*. Additional file 1: Figure S6. Significant pathways obtained from IPA Canonical Pathway Analysis (Ingenuity® Systems, www.ingenuity.com). Filled symbols represent genes associated with DMRs. a) Enrichment of hypermethylated MEF *Tdg-/-* DMRs associated with Wnt signaling pathway. 95 of 172 genes associated with DMR (BH corrected p-value = 4.15E-11), b) Enrichment of hypermethylated MEF *Tdg-/-* DMRs associated with embryonic stem cell pluripotency pathway (human). 78 of 153 genes associated with DMR (BH corrected p-value = 8.57E-11). Additional file 1: Table S1. 18 mouse methylomes, representing 6 biological cohorts, were generated using PE MeDIP-seq. Additional file 1: Table S2. Between replicate MeDIP-seq correlations, generated by QCSeqs, for the methylomes. Additional file 1: Table S3. Summarised output from GREAT analysis (McLean *et al.* 2010). a) Hypermethylated in MEF *Tdg-/-*, b) Hypomethylated in MEF *Tdg-/-*. Click here for file
