# Supplementary material for: Resources for methylome analysis suitable for gene knockout studies of potential epigenome modifiers
Source: Gigascience. 2012 Jul 12;1:3. doi: 10.1186/2047-217X-1-3 (PMC3617451; doi:10.1186/2047-217X-1-3)
Supplement: Additional file 1 Figure S1. — Example of saturation and coverage analysis, performed in MeDUSA using the MEDIPS bioconductor package. a) Saturation analysis for ESC1 b) Coverage analysis for ESC1. Additional file 1: Figure S2. Methylomes available through Ensembl (Flicek et al. 2011) as part of the EU project HEROIC. Additional file 1: Figure S3. Boxplots displaying a) the DMR size (bp) and b) the genomic distance between DMRs across different cohort comparisons. In b) the width of each box represents the relative number of DMRs in the comparison. Additional file 1: Figure S4: Read density (RPM) at DMRs found between NPC and ESC cohorts. MEF RPM also shown for these sites. Additional file 1: Figure S5. Read density (RPM) at DMRs between a) MEF Tdg+/+ and MEF Tdg--‒/--‒ b) NPC Tdg+/--‒and NPC Tdg--‒/--‒. Additional file 1: Figure S6. Significant pathways obtained from IPA Canonical Pathway Analysis (Ingenuity® Systems, www.ingenuity.com). Filled symbols represent genes associated with DMRs. a) Enrichment of hypermethylated MEF Tdg-/- DMRs associated with Wnt signaling pathway. 95 of 172 genes associated with DMR (BH corrected p-value = 4.15E-11), b) Enrichment of hypermethylated MEF Tdg-/- DMRs associated with embryonic stem cell pluripotency pathway (human). 78 of 153 genes associated with DMR (BH corrected p-value = 8.57E-11). Additional file 1: Table S1. 18 mouse methylomes, representing 6 biological cohorts, were generated using PE MeDIP-seq. Additional file 1: Table S2. Between replicate MeDIP-seq correlations, generated by QCSeqs, for the methylomes. Additional file 1: Table S3. Summarised output from GREAT analysis (McLean et al. 2010). a) Hypermethylated in MEF Tdg-/-, b) Hypomethylated in MEF Tdg-/-. [file 2047-217X-1-3-S1.PDF]

Supplementary Material

Supplementary Figure 1

Example of saturation and coverage analysis, performed in MeDUSA using the MEDIPS bioconductor package. a) Saturation analysis for ESC1 b) Coverage analysis for ESC1.

a	b
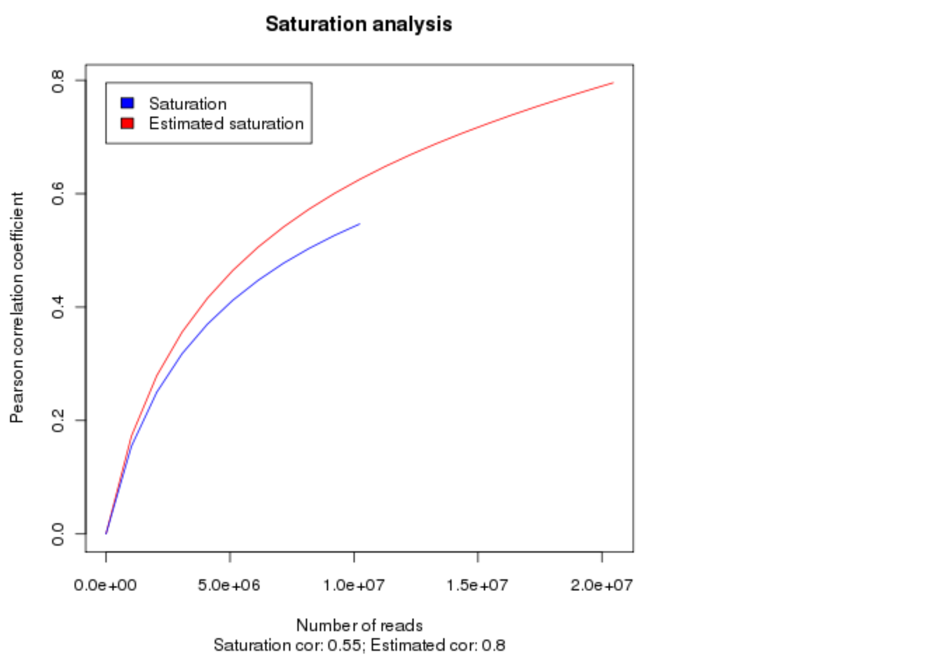	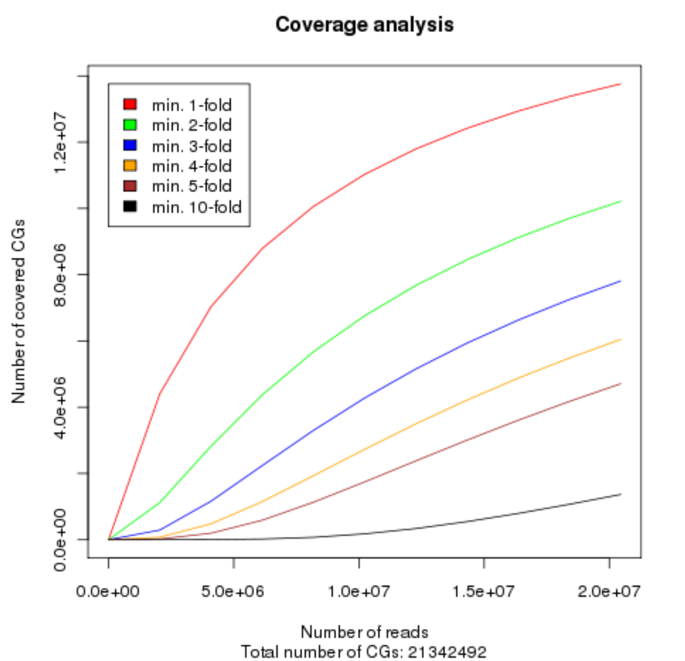

Supplementary Figure 2

Methylomes available through Ensembl (Flicek et al. 2011) as part of the EU project HEROIC.

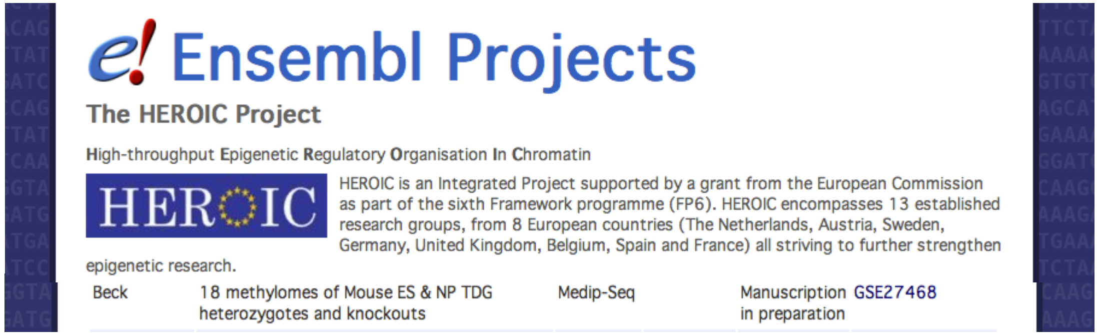

Supplementary Figure 3

Boxplots displaying a) the DMR size (bp) and b) the genomic distance between DMRs across different cohort comparisons. In b) the width of each box represents the relative number of DMRs in the comparison.

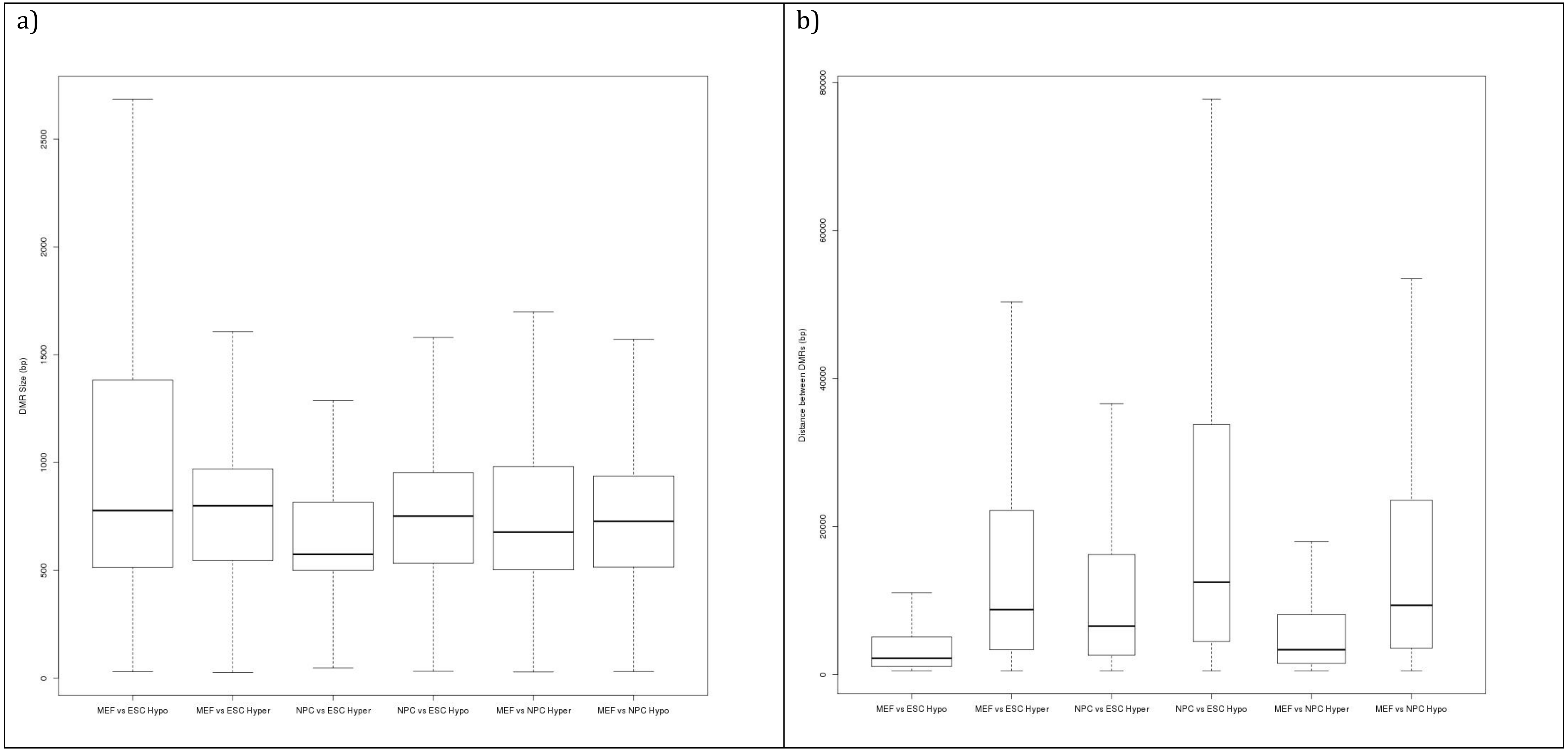

Supplementary Figure 4

Read density (RPM) at DMRs found between NPC and ESC cohorts. MEF RPM also shown for these sites.

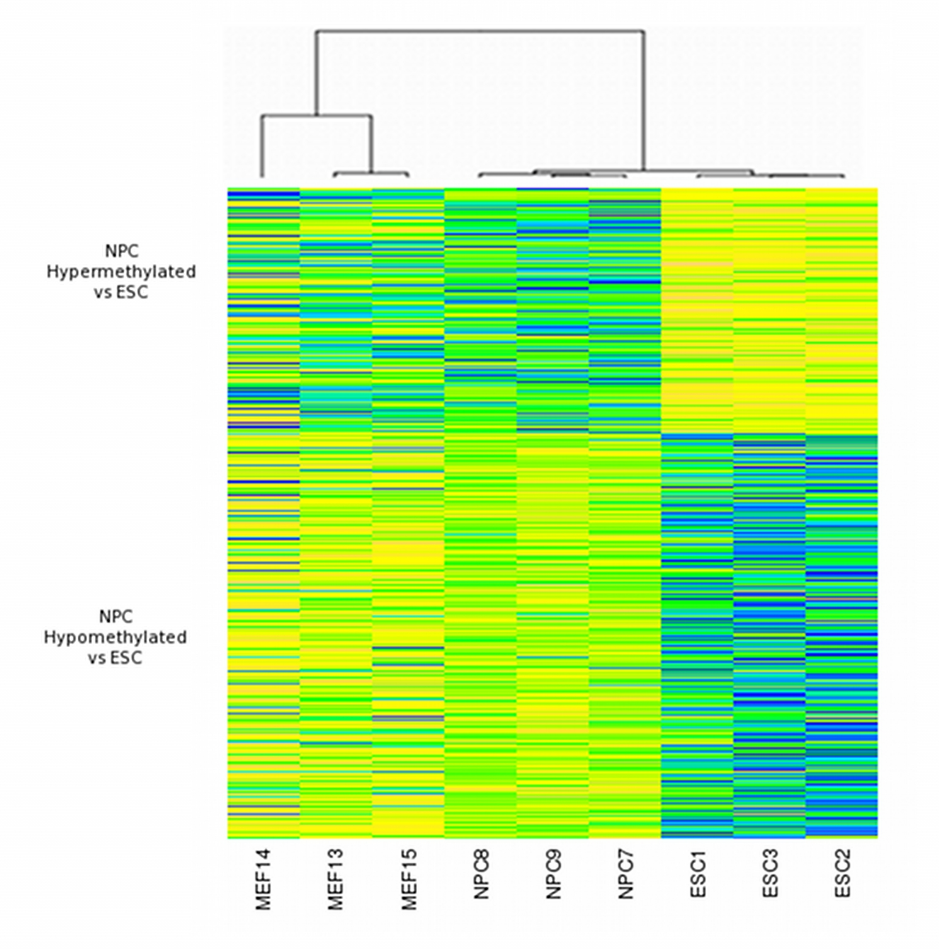

Supplementary Figure 5

Read density (RPM) at DMRs between a) MEF $Tdg^{+/+}$ and MEF $Tdg^{-/-}$ b) NPC $Tdg^{+/-}$ and NPC $Tdg^{-/-}$

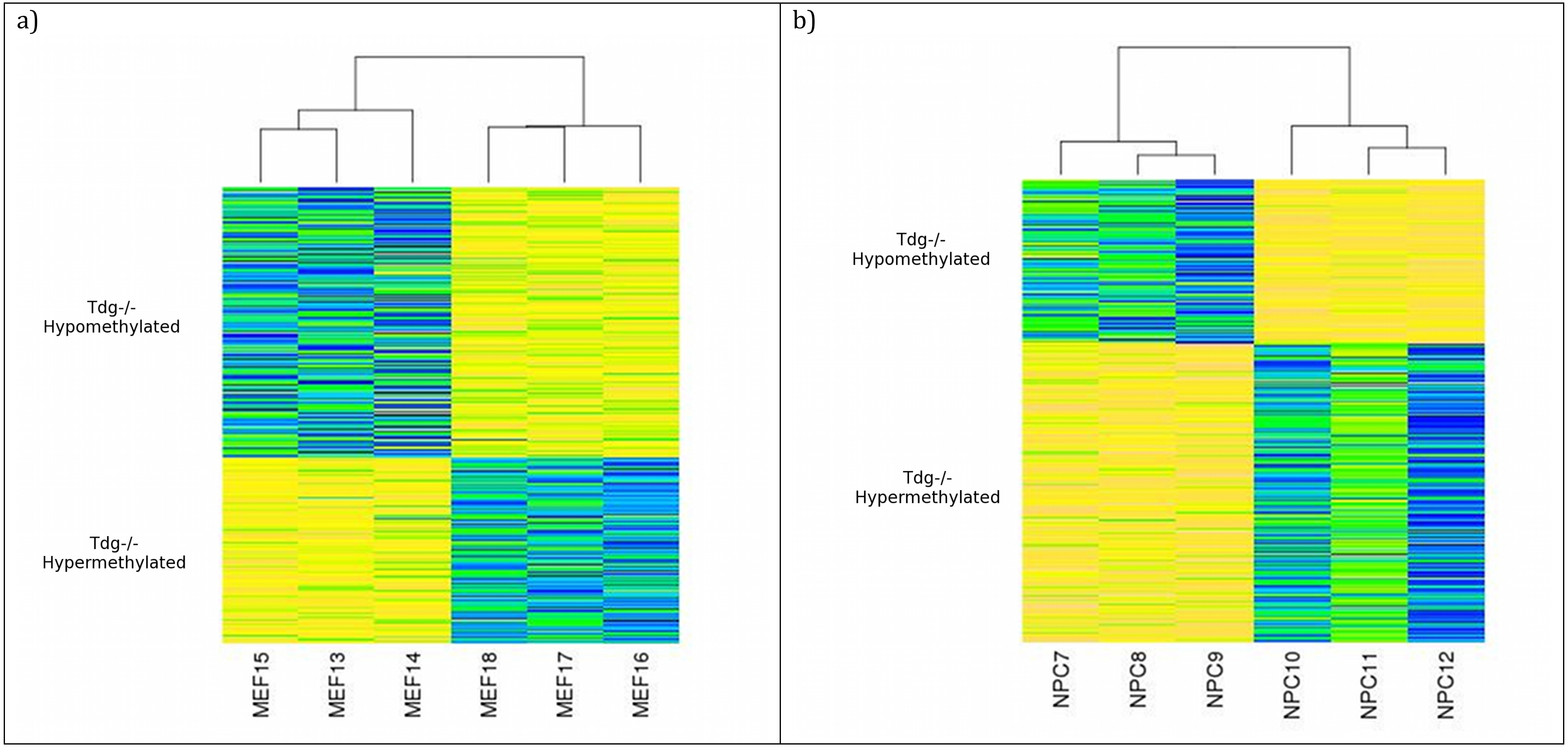

Supplementary Figure 6

Significant pathways obtained from IPA Canonical Pathway Analysis (Ingenuity® Systems, www.ingenuity.com). Filled symbols represent genes associated with DMRs. a) Enrichment of hypermethylated MEF *Tdg*$^{-/-}$ DMRs associated with Wnt signaling pathway. 95 of 172 genes associated with DMR (BH corrected p-value = 4.15E-11), b) Enrichment of hypermethylated MEF *Tdg*$^{-/-}$ DMRs associated with embryonic stem cell pluripotency pathway (human). 78 of 153 genes associated with DMR (BH corrected p-value = 8.57E-11).

a

Wnt/β-catenin Signaling

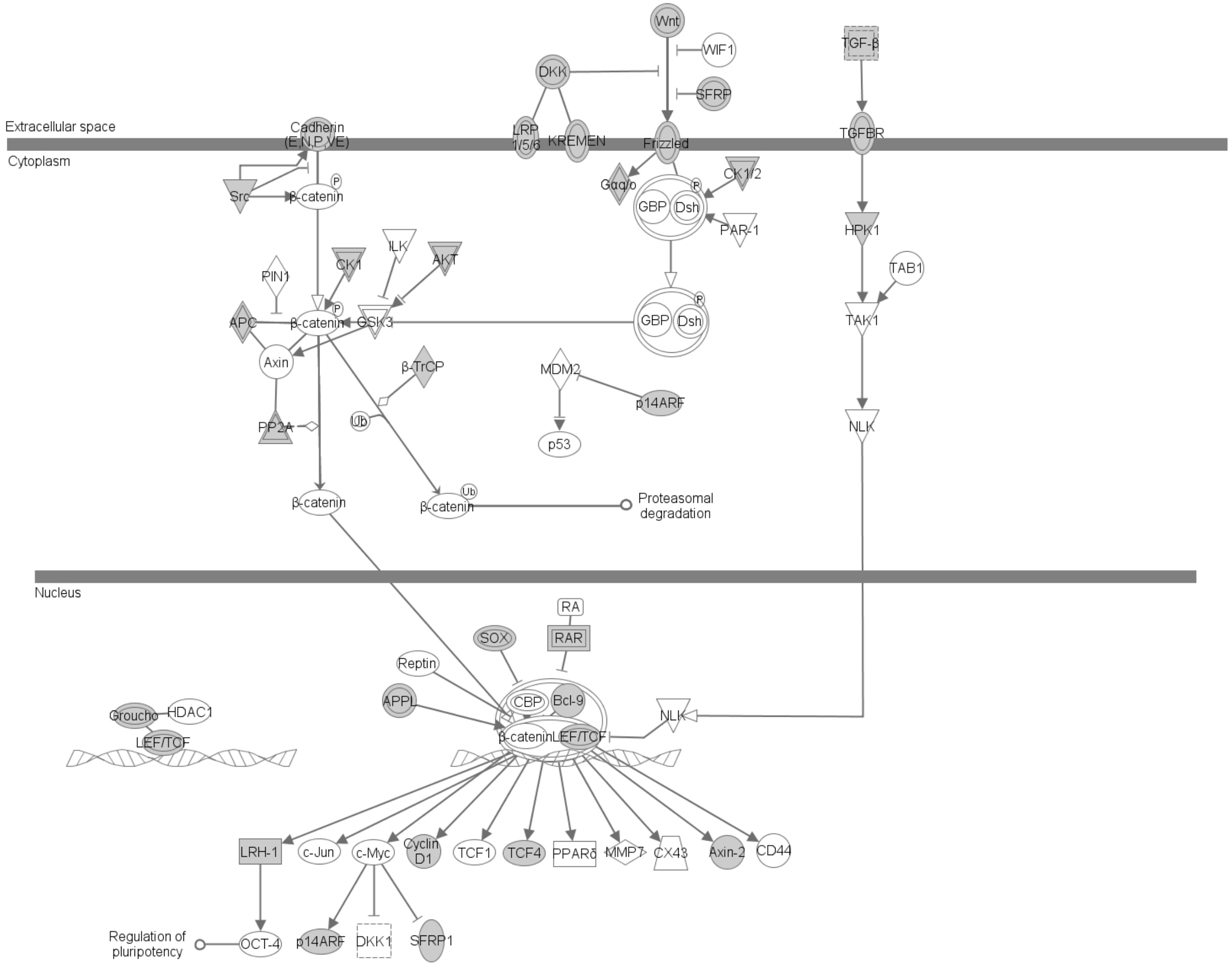

© 2000-2011 Ingenuity Systems, Inc. All rights reserved.

b

Human Embryonic Stem Cell Pluripotency

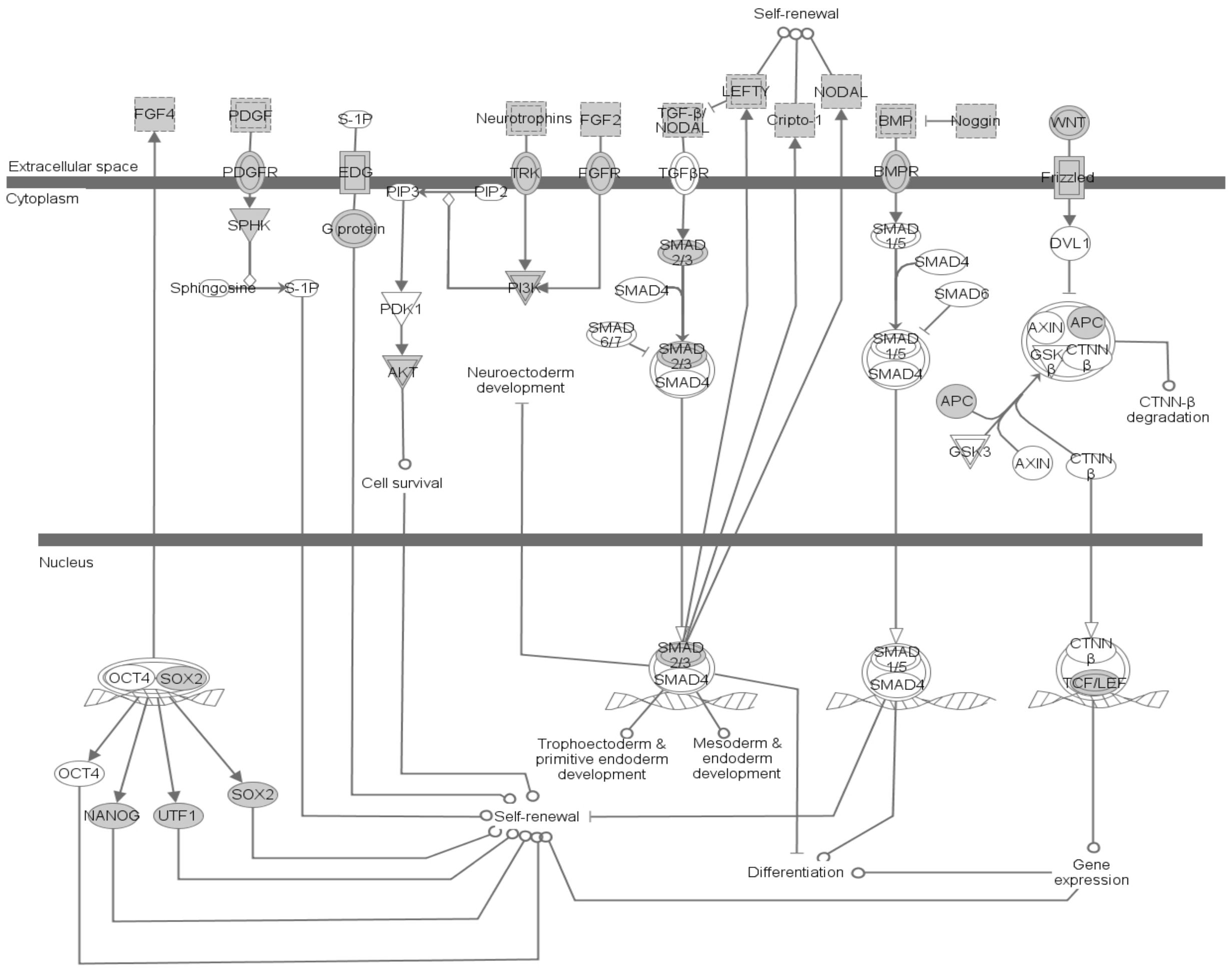

© 2000-2011 Ingenuity Systems, Inc. All rights reserved.

Supplementary Table 1

18 mouse methylomes, representing 6 biological cohorts, were generated using PE MeDIP-seq

Sample	Genotype	Cycles	Total Reads (pre-alignment)	Unique fragments (paired reads)	Mean Insert Size	CpG Enrichment Score	CpG coverage	%GC
**ESC1**	*Tdg*$^{+/-}$	36	62845000	20341082	151.78	2.29	65.20%	45
**ESC2**	*Tdg*$^{+/-}$	36	62364152	10709640	149.15	2.51	49.13%	48
**ESC3**	*Tdg*$^{+/-}$	36	54406944	14817391	141.17	2.57	54.99%	48
**ESC4**	*Tdg*$^{-/-}$	36	66927428	17614895	173.89	2.54	59.02%	49
**ESC5**	*Tdg*$^{-/-}$	36	65322058	14589787	167.09	2.48	55.27%	48
**ESC6**	*Tdg*$^{-/-}$	36	44856148	15827404	143.37	2.49	58.42%	47
**NPC7**	*Tdg*$^{+/-}$	36	45656784	15525293	158.85	3.11	54.44%	48
**NPC8**	*Tdg*$^{+/-}$	36	50774190	17196263	153.37	3.03	56.40%	48
**NPC9**	*Tdg*$^{+/-}$	36	67152858	16996748	147.37	3.16	53.65%	48
**NPC10**	*Tdg*$^{-/-}$	36	66567040	17297064	153.99	3.00	54.88%	47
**NPC11**	*Tdg*$^{-/-}$	36	69551064	21441777	152.98	3.10	58.85%	48
**NPC12**	*Tdg*$^{-/-}$	36	72155140	23156257	165.49	3.00	60.72%	48
**MEF13**	*Tdg*$^{+/+}$	36	47189760	9409098	149.91	3.91	34.12%	44
**MEF14**	*Tdg*$^{+/+}$	36	16940284	3160066	131.32	4.6	17.39%	45
**MEF15**	*Tdg*$^{+/+}$	36	32206682	6621484	163.02	3.81	30.55%	44
**MEF16**	*Tdg*$^{-/-}$	36	40284500	8645926	147.21	4.59	32.47%	45
**MEF17**	*Tdg*$^{-/-}$	36	45055076	9349976	153.37	4.47	34.00%	44
**MEF18**	*Tdg*$^{-/-}$	36	42421696	8794422	147.32	4.51	32.63%	45

Supplementary Table 2

Between replicate MeDIP-seq correlations, generated by QCSeqs, for the methylomes

	ESC1	ESC2	ESC3	ESC4	ESC5	ESC6	NPC7	NPC8	NPC9	NPC10	NPC11	NPC12	MEF13	MEF14	MEF15	MEF16	MEF17	MEF18
**ESC1**		0.53	0.51	0.55	0.52	0.54												
**ESC2**	0.53		0.63	0.57	0.56	0.63												
**ESC3**	0.51	0.63		0.55	0.54	0.66												
**ESC4**	0.55	0.57	0.55		0.61	0.56												
**ESC5**	0.52	0.56	0.54	0.61		0.54												
**ESC6**	0.54	0.63	0.66	0.56	0.54													
**NPC7**								0.83	0.84	0.83	0.87	0.85						
**NPC8**							0.83		0.85	0.83	0.86	0.82						
**NPC9**							0.84	0.85		0.84	0.88	0.83						
**NPC10**							0.83	0.83	0.84		0.87	0.87						
**NPC11**							0.87	0.86	0.88	0.87		0.87						
**NPC12**							0.85	0.82	0.83	0.87	0.87							
**MEF13**														0.90	0.92	0.76	0.77	0.77
**MEF14**													0.90		0.91	0.75	0.76	0.77
**MEF15**													0.92	0.91		0.73	0.75	0.75
**MEF16**													0.76	0.75	0.73		0.94	0.94
**MEF17**													0.77	0.76	0.75	0.94		0.94
**MEF18**													0.77	0.77	0.75	0.94	0.94	

Supplementary Table 3

Summarised output from GREAT analysis (McLean et al. 2010). a) Hypermethylated in MEF *Tdg*$^{-/-}$, b) Hypomethylated in MEF *Tdg*$^{-/-}$.

a)

Ontology	Term Name	Binom FDR Q-Val	Hyper FDR Q-Val
**GO Molecular Function**	transcription regulator activity	0	2.57E-37
**GO Molecular Function**	DNA binding	0	2.12E-21
**GO Molecular Function**	nucleic acid binding	0	5.22408E-05
**GO Molecular Function**	transcription factor activity	1.2257e-319	6.43E-39
**GO Molecular Function**	sequence-specific DNA binding	1.765e-319	1.59E-36
**GO Biological Process**	system development	0	4.82E-66
**GO Biological Process**	multicellular organismal development	0	2.58E-66
**GO Biological Process**	anatomical structure development	0	1.20E-62
**GO Biological Process**	developmental process	0	9.61E-63
**GO Biological Process**	organ development	0	3.50E-48
**GO Biological Process**	regulation of metabolic process	0	4.90E-29
**GO Biological Process**	regulation of cellular metabolic process	0	6.33E-29
**GO Biological Process**	regulation of primary metabolic process	0	2.98E-26
**GO Biological Process**	regulation of nucleobase, nucleoside, nucleotide and nucleic acid metabolic process	0	4.70E-23
**Mouse Phenotype**	nervous system phenotype	0	7.21E-80
**Mouse Phenotype**	abnormal nervous system morphology	6.50E-299	1.46E-55
**Mouse Phenotype**	lethality-prenatal/perinatal	1.34E-271	2.84E-34
**PANTHER Pathway**	Wnt signaling pathway	1.52E-33	8.37E-07
**MSigDB Pathway**	Pathways in cancer	3.01E-45	1.38E-12
**MSigDB Pathway**	MAPK signaling pathway	4.81E-30	1.21445E-06

**MSigDB Pathway**	Basal cell carcinoma	8.21E-29	9.02E-08
**MSigDB Pathway**	Wnt signaling pathway	1.79E-25	1.26463E-06
**MSigDB Pathway**	Genes related to Wnt-mediated signal transduction	1.90E-24	2.44941E-05
**MGI Expression: Detected**	TS20_organ system	0	3.18E-66
**MGI Expression: Detected**	TS20_embryo	0	3.26E-65
**MGI Expression: Detected**	Theiler_stage_20	0	8.15E-62
**MGI Expression: Detected**	TS17_embryo	0	9.61E-55
**MSigDB Perturbation**	Set 'H3K27 bound': genes posessing the trimethylated H3K27 (H3K27me3) mark in their promoters in human embryonic stem cells, as identified by ChIP on chip.	0	1.75E-158
**MSigDB Perturbation**	Set 'Eed targets': genes identified by ChIP on chip as targets of the Polycomb protein EED [Gene ID=8726] in human embryonic stem cells.	0	9.87E-121
**MSigDB Perturbation**	Set 'Suz12 targets': genes identified by ChIP on chip as targets of the Polycomb protein SUZ12 [Gene ID=23512] in human embryonic stem cells.	0.00E+00	3.20E-136
**MSigDB Perturbation**	Set 'PRC2 targets': Polycomb Repression Complex 2 (PRC) targets; identified by ChIP on chip on human embryonic stem cells as genes that: posess the trimethylated H3K27 mark in their promoters and are bound by SUZ12 [Gene ID=23512] and EED [Gene ID=8726] Polycomb proteins.	1.11E-281	3.17E-107

b)

Ontology	Term Name	Binom FDR Q-Val	Hyper FDR Q-Val
**GO Molecular Function**	cation channel activity	1.97E-46	8.42E-13
**GO Molecular Function**	voltage-gated ion channel activity	1.36E-45	2.80E-08
**GO Molecular Function**	voltage-gated cation channel activity	5.04E-36	2.13825E-06
**GO Molecular Function**	potassium channel activity	1.79E-27	1.61838E-06
**GO Biological Process**	extracellular structure organization	1.22E-38	0.000483757
**GO Biological Process**	potassium ion transport	1.07E-28	3.05244E-06
**GO Biological Process**	divalent metal ion transport	1.98E-28	0.000494935
**MGI Expression: Detected**	TS17_surface ectoderm	6.96E-53	2.06E-11

**MGI Expression: Detected**	TS17_embryo;ectoderm	3.38E-50	2.06E-11
**MGI Expression: Detected**	TS17_urogenital system	3.86E-40	9.21E-10
